# “Engaging in my rural community”: perceptions of people aged 85 years and over

**DOI:** 10.1080/17482631.2018.1503908

**Published:** 2018-08-13

**Authors:** Stephen Neville, Jeffery Adams, Sara Napier, Kay Shannon, Debra Jackson

**Affiliations:** a Department of Nursing, Auckland University of Technology, Auckland, New Zealand; b SHORE & Whariki Research Centre, Massey University, Auckland, New Zealand; c Oxford Institute of Nursing, Midwifery & Allied Health Research, Oxford Brookes University, Oxford, UK

**Keywords:** Physical environment, social engagement, environmental gerontology, older adults, age-friendly communities, rural ageing

## Abstract

**Purpose: **Globally, numbers of people aged 85 years and over are increasing. Many older people, including those 85 years and over, are ageing in rural areas. For successful ageing in place, physical and social environments must be appropriate. The aim of this study is to understand the influence the physical and social environments have on enabling those aged 85 years and over to remain engaged in a rural community. **Method: **Utilizing an environmental gerontological approach, semi-structured interviews were undertaken with 15 people who lived independently in a rural community. Following transcription data were analyzed and themes identified. **Results:** Two themes were identified; “Negotiating the physical environment: ‘Getting there and back’” and “Maintaining social networks: ‘Places to go, people to see’”. The findings provide insight into the importance of driving, parking close to amenities and negotiating the local environment to this group of older people and their ability to engage with their community. All participants agreed social engagement with friends, family or neighbors was important to them. **Conclusion: **These findings highlight the interconnection between physical and social environments. An enabling physical environment is essential to support social participation of people aged 85 years and over.

## Introduction

The demographic characteristics of rural populations are changing, and as a result rural populations, particularly those in the western world, are typically older in comparison with urban areas (Neville, Napier, Adams, Wham, & Jackson, 2016). This can be partially explained by an exodus of younger people to cities in search of employment and an associated inward migration of older people to rural areas owing to their affordability, as well as for lifestyle choices and the desire to reconnect with rural roots (Stockdale, 2011; Vuin, Carson, Carson, & Garrett, 2016). Consequently, many older people are attracted to rural environments.

In many countries ageing in place is a central tenet of government initiatives to address the challenges of ageing populations and a catchphrase for policy-makers and health professionals. Ageing in place refers to older people being able to remain living in their community as they age, either with or without support, as opposed to relocating to a care facility or living elsewhere (Wiles, Leibing, Guberman, Reeve, & Allen, 2012). Older people living in rural areas often develop strong attachments to their communities with research showing they expect to remain living rurally as they age (Dye, Willoughby, & Battisto, 2010). Ageing in place relies on communities being appropriate places for older people to age in, whether that be in urban or rural environments.

Those aged 85 years and over, also referred to as the oldest-old, are the fastest-growing older adult group with increasing numbers living independently in their communities (Neville, Russell, Adams, & Jackson, 2016). Age-related physical and social challenges exist for many people in this age group, including reduced energy levels, mobility and social opportunities (Alley, Liebig, Pynoos, Banerjee, & Choi, 2007). Therefore, community physical and social environments must facilitate and support the oldest-old to successfully age in place.

The World Health Organization (WHO) developed the “Global Age-Friendly Cities: A Guide” for use in urban environments (World Health Organisation, 2007). This has since been adapted for use in rural and remote areas (Federal Provincial Territorial Ministers Responsible for Seniors, 2007). Both of these documents are available to guide local government and other agencies to ensure their communities support ageing in place for all older adults.

The cornerstone of age-friendly initiatives is ensuring the physical environment, including the availability of transportation, is conducive to enabling older people to easily navigate their community. By doing so social participation is fostered and supported (Neville, Napier, et al., 2016). Research has shown that rural communities with high walkability are associated with independence and increased social interactions (Bacsu et al., 2014). Other studies have revealed difficulties accessing public transport services and the heavy reliance on owning a car, and being able to drive, as a barrier for older people living in rural environments to maintain friendships and keep in touch with significant others (Walker et al., 2013; Walsh, O’Shea, Scharf, & Murray, 2012). Social networks typically consist of family, friends and neighbours (Cornwell, Laumann, & Schumm, 2008). These networks provide not only social support but also practical support such as help around the house, as well as driving older people to appointments and to do shopping when public transport is not available or suitable.

Reduced opportunities for meaningful social engagement are linked to loneliness which in turn is correlated with increased age and is a predictor of a variety of physical and mental health concerns (Dahlberg, Andersson, McKee, & Lennartsson, 2015). Consequently, the health and well-being of older people living rurally can be influenced by both physical and social environments, including the ability to navigate their surroundings (Annear et al., 2014; Keating & Eales, 2013). However, government agencies and policy-makers do not always seek older people’s views, particularly from those aged 85 years and over, when planning physical environments or providing social services, including those in rural areas (Wiles et al., 2012). Consequently, policy and services are often formulated without experiential information provided by older people.

### Research aim

The aim of this research is to understand the influence the physical environment and social networks have on enabling the oldest-old to remain engaged within a rural community. Our specific focus is on the oldest-old living in rural settings, as there are few international studies that exclusively focus on this group. This study is particularly relevant in New Zealand, as age-friendly initiatives are recent and only focused on urban areas.

## Methods

### Theoretical orientation

This qualitative study is informed by environmental gerontology to better allow us to address the research aim of exploring the influences the physical environment has on enabling people aged 85 years and over to remain engaged within a rural community. This theoretical orientation is grounded in published works derived from the ecology of ageing and Lawton’s influential person–environment fit model (Lawton & Nahemow, 1973; Wahl, Iwarsson, & Oswald, 2012). Environmental gerontology provides a framework from which to explain the interaction between older adults and their physical and social environments (Schwarz, 2012). The underlying supposition inherent in environmental gerontological thinking is that ageing makes older people vulnerable to their environments which impacts on health and well-being (Schwarz & Scheidt, 2013). The present study has utilized the central principles of environmental gerontology to explore the influence the physical environment has on this group of older people’s physical and social engagement with their local rural community.

### Design

We considered each of the domains of the COnsolidated criteria for Reporting Qualitative research when developing and reporting the methods and findings of this study (Tong, Sainsbury, & Craig, 2007).

A qualitative descriptive approach, underpinned by the philosophical position of naturalistic inquiry, was utilized in this study. Qualitative description is an appropriate design to use for an exploratory study when discovery of the who, what and where and straightforward descriptions of experiences are required; thus, this approach was considered suitable to undertake this study of an under-researched and emerging topic (Sandelowski, 2000).

### Sampling and recruitment of participants

In this study, a volunteer sampling process was deployed. It was promoted via an advertisement and an editorial piece in the local newspaper and in a returned services association newsletter, as well as posters strategically placed on community noticeboards. Interested potential participants made telephone contact with a member of the research team. Participants who met the inclusion criteria were recruited into the study. Inclusion criteria were people aged 85 years and older, who lived independently in the surrounding rural area and accessed services and activities in the local rural town. Additionally, participants would be required to participate in an interview of approximately 1 h. Ten participants were recruited from advertising, and a further five participants were recruited through a third party who was well connected to this community. The study was explained over the phone, and if potential participants met the inclusion criteria and were still interested in participating, a convenient time and place to meet and undertake the interview were negotiated. A respectful and empathetic approach and contacting the participants again the day before the interview helped to build trust. Table I provides a demographic profile of participants.

**Table I.** Demographic profile of participants.

Pseudonym	Gender	Age	Relationship status	Years in area
Jean	Female	87	Widowed	53
Annie	Female	85	Divorced	5
James	Male	86	Married	86
Ted	Male	90	Widowed	45
Beth	Female	85	Widowed	0.5
Martin	Male	92	Widowed	26
Ruth	Female	87	Widowed	30
Frank	Male	87	Widowed	20
David	Male	93	Married	29
Sylvia	Female	90	Widowed	37
Esme	Female	85	Married	12
Harriet	Female	90	Widowed	33
Emily	Female	86	Widowed	15
Rose	Female	87	Widowed	23
Robert	Male	92	Married	32

### Setting

The study was undertaken in a small rural town with a population of 3,909 at the 2013 census. Approximately 26% of the population is aged 65 years and over (Statistics New Zealand, 2013). The town is a service centre for the local largely farming and retirement communities. It has a range of services including supermarkets, cafes, banks, retail, library, information centre, residential care facilities and primary health services.

### Data collection and analysis

Before undertaking the interview, all participants were provided the opportunity to read the information sheet and have any questions answered. At this point, the consent form was completed. Digitally recorded semi-structured in-depth interviews were then undertaken with 15 people who met the inclusion criteria in 2015. All participants preferred to complete the interview in their own home with the length of interviews ranging from 45 to 90 min. Two of the participants chose to have their partners present. Recruitment and interviewing continued until data saturation was reached, and no new significant ideas were identified.

The interview data were transcribed verbatim by a professional transcriber who had signed a confidentiality agreement. Following transcription, a thematic data-analytic process was undertaken utilizing Braun and Clarke (2006) six-phased framework. The first phase involved three researchers reading all transcripts several times to gain a thorough understanding of the content. This included reading the transcripts line by line to develop an initial list of codes that captured the essence of the ideas presented. Common patterns evident across codes were then identified and agreed on before being combined to form initial themes. Phase 4 involved checking that each theme had sufficient supporting data followed by the development of a thematic map containing themes and subthemes. The final phase in the data-analytic process involved attaching illustrative verbatim excerpts from the data. These were checked by all members of the research team. Data were analysed using a semantic approach, with the themes identified from the explicit or surface meanings of the data. A summary of the findings was presented in a well-attended community forum. Recommendations were then developed and an agreed future research plan determined.

### Ethics

Ethical approval for this study was obtained from the Auckland University of Technology Ethics Committee (AUTEC 15/100) and Massey University Human Ethics Committee (MUHECN 15/010). The potential vulnerability of this age group was acknowledged; however, no major ethical concerns were identified. Integral to all aspects of the study were the ethical principles of justice, beneficence and respect for personal integrity and human vulnerability (Woods & Lakeman, 2016). These were adhered to throughout the study. Pseudonyms are used to ensure anonymity of participants.

## Results

This article focuses on presenting two themes identified in the data relevant to the research aim. The themes are: (a) negotiating the physical environment: “getting there and back”, and (b) social networks: “places to go, people to see”.

### Negotiating the physical environment: “getting there and back”

This theme captures the importance recognized by all participants that transportation was essential to being able to engage with their community. Driving a privately owned vehicle was the most common mode of transport used by this group.

Thank goodness I am still able to drive. If I want to go somewhere or visit somebody, well I hop in the car and go … . (Frank, 87 years)

Participants greatly valued being able to drive. Because of this, not being able to drive in the future concerned them.

Somehow I’m beginning to think that in the future I may be isolated if I can’t get out and about. I won’t be able to join in the things I enjoy and could feel cut off. I’ve been alright so far but I’m lucky that I can still drive my car. If I couldn’t drive I’d have to consider living somewhere else. It’s always in the back of my mind, you know. We have got a retirement village here, but that doesn’t appeal to me [living there]. (Rose, 87 years)

Participants realized the importance of driving to their ability to live the life they wanted and to enjoy the activities they valued. They felt the ability to drive was central to being able to age in place. Without the ability to drive some participants identified they might need move to a retirement village.

If I couldn’t get around, I’d have to move. I’d probably move closer to my children and live in a retirement village. It won’t be ideal, I’d rather live here where my friends are but transport is a problem in rural areas like here. (Emily, 86 years)

A feature of the physical environment related to transport that had an influence on engagement with this community was having access to an appropriate place to park their car that allowed the required time to undertake tasks like shopping. Participants reported that they primarily moved about within their community at times when parking was easy to obtain.

I’m an early morning person so I always go to town early so I always get a car park. When I go to the supermarket you always get parking there because they have a parking garage so I always have plenty of time. When I go to the bank or whatever I always find a park, I never have any problems. (Frank, 87 years)

Equally, holding a mobility parking permit [providing access to dedicated parking spaces] enabled Rose (87 years) to park close to where she needed to go.

I’ve got a disability ticket [mobility parking permit] and that’s been marvellous, especially for grocery shopping. Generally, I’ll go to [name of the supermarket] and they’ve got a park just outside which means I can manage really well.

The dynamics of traffic flow meant driving around the town for some participants was difficult. A particularly complex intersection at one end of the main street was identified by several participants as being difficult to negotiate when driving. Several participants avoided this intersection by taking an alternative route.

I worked out that everyone has ten options at that junction, and one day I went to move and somebody came at me from the side. That scared the wits out of me. Normally I avoid this intersection completely and drive right to the top of the hill, and come through the traffic lights, rather than cut across. I treat that junction with great respect. (Esme, 85 years)

The intersection that Esme refers to separates a retirement village from the town. Several participants noted that older people living in the retirement complex struggled to negotiate this intersection and were concerned that this was a barrier to these people being able to engage with their community. This was so even for pedestrians.

There is no pedestrian crossing for those people from the retirement village to use. So … if you use a walking frame it is impossible to cross the road, no one will stop for them. We have complained about it for a while, nothing has been done about it. It’s a real problem for the oldies there. For those who do not drive … it’s impossible for them to go to town. (Annie, 85 years)

For those who did not drive there was a public bus service, and although limited, some older people made use of this mode of transport.

I use the swimming bus on a Friday that goes to [name of the town close by], I use that. Then I catch the bus to town twice a week to do my shopping and catch up with friends. (Beth, 85 years)

However, some participants identified barriers to using the bus service which ultimately negatively impacted on their ability to engage with their community. Difficulties with getting on and off the bus, particularly when carrying packages, were problematic for some participants,

If you go by bus to do your grocery shopping it means you’ve still got to carry all that shopping around and get it home. (Jean, 87 years)

Using public transport as a means to “getting out and about” is further complicated for some older people who have difficulty with particular issues associated with getting on and off the bus. Some buses had stairs that were difficult for participants to negotiate.

I find the buses have a lot of steps. They are difficult to negotiate, a bit like a mini Mount Everest. I don’t know who designed those steps. I understand maybe they’ve got to have steps, but they seem to me to be very awkward for an older person to navigate and stop me from getting out and about. (Ruth, 87 years)

### Maintaining social networks: “places to go, people to see”

This theme captured the importance of maintaining social networks, including friendships, for participants in this study.

When you get older, friends are what matter. Family are nice but they are all busy … your friends are your age and you understand one another. (Sylvia, 90 years)

The desire to maintain friendships was an important influencing factor on participants when they considered moving to a new location.

Shifting away is a problem to think about because you are losing a lot of friends and as you get older it’s not so easy to make friends … and I’ve got a good set of friends. (Rose, 87 years)

The development of strong relationships with neighbours was important for some participants, particularly those with no family living close by. They greatly appreciated the neighbourliness of others in connecting, engaging and offering assistance to them.

Where I live is very good. Over there is [name of person] who lost his wife six months ago. He’s a quiet gentle sort of person. Right next door I’ve got a lovely family, couple and four children and across the road is my friend. To me neighbours and friends are important … I’m on my own and at my age I like to know that there are people around who can keep an eye on me, help me if I need it. If my friend over the road sees me in the town she stops and gives me a lift back … I like that. (Ruth, 87 years)

Sometimes neighbours played the role of surrogate families for those with no family close by, assisting by providing care when some participants were unwell, while at the same time providing opportunities for ongoing engagement and social interaction.

The lady over the road looked after me when I got out of hospital. I was really grateful you know … she got me back on my feet, up and running again. So to say thanks to her I’m taking her on holiday for a fortnight … she’s over the moon about that … we’ll have a great time. (Ted, 90 years)

Ted’s experience supports the important role communities have in supporting others and providing opportunities for social engagement.

It’s a great community, everyone is there for each other … you look around and know who is not well and who needs help. Everybody looks out for each other. I have a good set of friends, people who live here are really friendly … it’s a really caring community. (Jean, 87 years)

Increasing migration both into and out of the neighbourhood changed the character of the community and negatively impacted on opportunities for social interaction. Rose comments on her new neighbours who are not around during the day and the impact this has on her feeling connected to her community.

… the neighbourhood is starting to change and we’ve got people who are both working. I’m thinking of two lots who have come in recently, both young and working. With them away during the day I feel a bit isolated, there isn’t the same opportunity to chat. That’s the sort of feeling I get. (Rose, 87 years)

Community groups such as Neighbourhood Watch also provided fora for enabling community connections, and contributing to creating a sense of belonging for older people. Participants who had only recently moved to the area identified that belonging to the Neighbourhood Watch Group was an important conduit that provided new residents with opportunities for engagement and community integration.

We are just settling in at the moment and trying to get to know the locals. The local Neighbourhood Watch Group contacted us inviting us to their meeting. We have been [to a meeting] where they made us feel welcome saying they were here for us … it’s very social you know. (Robert, 92 years)

## Discussion

This study explored the influence of the physical environment on physical and social engagement for a group of older, rural-dwelling people, aged 85 years and over. The first theme, “Negotiating the physical environment: ‘Getting there and back’” encompassed and highlighted some of the key issues and features of the physical environment impacting on their ability to effectively engage with their community.

The majority of participants had access to a private car either because they drove or owing to a spouse, family member or significant other who provided that service. These participants recognized driving as being central to their independence as well as their physical and social engagement with the community. This finding is consistent with other studies of rural older people, which have identified limited public transport options meaning a heavy reliance on driving was linked to independence and being engaged within their neighbourhoods (Van Dijk, Cramm, Van Exel, & Nieboer, 2015; Ward, Somerville, & Bosworth, 2013). Furthermore, driving allowed the spontaneity and control over discretionary outings associated with independent means of travel.

A complex intersection within the town became a physical barrier for some participants whether they were driving or walking, forcing some to take an alternative and longer route which was identified as a barrier to engagement. Well-designed physical environments that allow older people to easily negotiate whether walking or driving have been identified as essential components supporting community engagement (O’Brien, 2014). The importance of good environmental design is also supported by Van Dijk et al. (2015) who asserted that supportive communities provide an environment conducive to older adult’s engagement regardless of physical and/or social abilities.

Issues with parking including a shortage of parking spaces and short time limits were problematic for several participants, interfering with their community engagement activities. Some of these activities included essential services like shopping and banking. The walkability of the local environment also identified as being important to this group of participants. These findings resonate with other studies that have identified that a lack of parking, parking time limits and parking not located close to services interfered with older people’s community engagement (Novac & Menec, 2014).

Not all participants had access to a car and public transport operations were minimal. Flexible bus services involving door-to-door pick-up and drop-off have been found to increase age-friendliness of public transport in rural areas (Broome, Worrall, Fleming, & Boldy, 2012). Our participants also highlighted the inaccessibility of the buses, and linked number of stairs required to get on and off the bus as being a factor in the ability and willingness to use public transport. Furthermore, in the present study, the separation of the retirement village from the town by a main highway and limited public transport options restricted opportunities for physical and social engagement. Perceived concerns related to driving ability in the future and limited alternative transport options meant participants questioned their ability to age in place.

The second theme, “Maintaining social networks: ‘Places to go, people to see’” related to the importance of the social environment. Participants reinforced the importance of family, friends and neighbours in enabling those aged 85 years and over to remain socially engaged with their community. In the present study, most participants had developed enduring and satisfying social networks, as well as being actively involved in community groups, and as such felt connected to their community. Changes in social networks affect social engagement in communities.

Findings from the present study identified that although family were acknowledged as important, having an established social network of friends and neighbours was more highly regarded. Needing support from family was balanced with living in a familiar community with an established social network of friends and neighbours. Social networks consisting of friends rather than family has been shown to contribute to resilience in older people, particularly those in the oldest-old age group living in rural communities (Wells, 2009). This is particularly salient when the reality for many older people living rurally is their families do not live close by (Stockdale, 2011).

Social changes to neighbourhoods impacted on opportunities for social engagement in this group. For example, living in a street comprised predominantly of younger working age people reduced avenues for spontaneous social engagement during the day as they were only at home later at night and on the weekends. As people age, they tend to spend more time at home and in their neighbourhoods (Gardner, 2011). Research in rural areas of Ireland (Walsh et al., 2012) found that older people depended on neighbours to be there when needed, as also evident in the findings of this study.

The availability of community groups, for example Neighbourhood Watch, represented opportunities for those aged 85 years and over to attend and engage with others in the community, and, as such, supported being socially engaged. Groups such as Neighbourhood Watch are voluntary organizations that require leaders, to organize and publicize, and volunteers to host and distribute information. To ensure the longevity of rural community groups that provide opportunities for social engagement, succession planning for leaders and volunteers is crucial (Winterton, Clune, Warburton, & Martin, 2014).

The findings from this study have been presented at a public forum in the community where data were collected. This forum was extremely well attended and resulted in a clear mandate from those present to continue to work with the community to plan and undertake future research. A key limitation of this project was the homogeneity of the research participants who all identified as New Zealand European. Consequently, the views of older Māori (the indigenous people of New Zealand) and older people from other ethnic groups are missing. This was also highlighted in the community forum. The next stage of this research programme will include a heterogeneous group of older people that transcends ethnicity to also include those from a variety of sociocultural groups, for example older people living with a disability.

## Conclusion

The results of this study highlight the relationship between the physical environment and continued engagement in community for this group of oldest-old rural-dwelling people. An enabling physical environment is essential to support community and social participation of oldest-old people, who continue to contribute to civic life via voluntary organizations. This research has added the voice of oldest-old people living rurally to the existing recommendations for the development of age-friendly communities. Policy-makers and planners will find this information useful as they prepare to cater for the increasing numbers of oldest-old people in the population.
